# Analysis and prediction of improved SEIR transmission dynamics model: taking the second outbreak of COVID-19 in Italy as an example

**DOI:** 10.3389/fpubh.2023.1223039

**Published:** 2023-08-24

**Authors:** Ming Lu, Xu-yang Zheng, Wei-nan Jia, Chun-zhi Tian

**Affiliations:** Information Engineering College, Wenzhou Business College, Wenzhou, China

**Keywords:** SEIR model, prediction, machine learning, transmission dynamics, anti-epidemic

## Abstract

This study aimed to predict the transmission trajectory of the 2019 Corona Virus Disease (COVID-19) and analyze the impact of preventive measures on the spread of the epidemic. Considering that tracking a long-term epidemic trajectory requires explanatory modeling with more complexities than short-term predictions, an improved Susceptible-Exposed-Infected-Removed (SEIR) transmission dynamic model is established. The model depends on defining various parameters that describe both the virus and the population under study. However, it is likely that several of these parameters will exhibit significant variations among different states. Therefore, regression algorithms and heuristic algorithms were developed to effectively adapt the population–dependent parameters and ensure accurate fitting of the SEIR model to data for any specific state. In this study, we consider the second outbreak of COVID-19 in Italy as a case study, which occurred in August 2020. We divide the epidemic data from February to September of the same year into two distinct stages for analysis. The numerical results demonstrate that the improved SEIR model effectively simulates and predicts the transmission trajectories of the Italian epidemic during both periods before and after the second outbreak. By analyzing the impact of anti-epidemic measures on the spread of the disease, our findings emphasize the significance of implementing anti-epidemic preventive measures in COVID-19 modeling.

## 1. Introduction

The COVID-19 pandemic has emerged as a significant global public health crisis, causing severe inflammation of the lungs due to a type of coronavirus. The virus’s high transmission rate, severe infection outcomes, and unpredictable epidemic timeline have posed an ongoing threat to human health, causing significant damage to the global economy. As stated by WHO Director-General Dr. Tam Desai, this pandemic is a once-in-a-century health crisis, and its ramifications will last for decades. Therefore, understanding the epidemic’s spreading mechanism (1, 2), analyzing the impact of anti-epidemic measures on its spread (3–5), and predicting its development trend and turning point have become critical issues (6–8).

Since the outbreak of COVID-19, modeling and analyzing the spread of the disease has become a significant area of focus. Common infectious disease models can be classified into SI, SIS, SIR, SIRS, and SEIR models depending on the specific disease’s characteristics. While some researchers have used the SIR model to analyze the epidemic situation in various countries (9–11), it is worth noting that COVID-19 patients have an exposed period and are infectious, making the SEIR model a more appropriate choice for analysis. Cao (12) developed an enhanced SEIR model that incorporated measures such as medical tracking and quarantine, based on limited data available in the early stages of the COVID-19 outbreak. In Wang’s model (13), infected individuals were classified into symptomatic and asymptomatic categories, and a network model was established to predict when Wuhan and its surrounding areas could resume economic activities. Tang (14) incorporated the contact rate and diagnosis rate dynamics into their model parameters. Li (15) estimated the proportion of symptomatic and asymptomatic infected individuals in China before January 23, as well as the ratio of symptomatic to asymptomatic infections. Zhang (16) introduced time-lag components and established a multi-stage time-delay dynamic model to investigate the impact of transportation on COVID-19 transmission. Zhong (17) developed a novel coronavirus pneumonia model based on transportation system dynamics and concluded that transportation has a positive feedback effect on the spread of COVID-19. Bag (18) utilized spatial statistical analysis to investigate the temporal and spatial patterns of COVID-19 transmission in India. Some researchers have employed advanced deep learning algorithms to construct artificial intelligence models for predicting the epidemic’s development (19–21). However, classical SEIR model parameters are fixed and may not accurately reflect the current epidemic situation’s evolving trend. As a result, due to changes in government anti-epidemic measures, improvements in medical care, and advancements in detection capabilities over time, dynamic model parameters are essential (22).

Through extensive research and analysis of the current SEIR model based on COVID-19, it has been found that while most studies are capable of short-term predictions within a specified period (23, 24), there is a lack of effective and accurate long-term prediction and anti-epidemic models. To address this issue, this study takes a comprehensive approach by considering various factors such as epidemic transmission characteristics (25), intervention measures (26), detection capabilities, and others, to enhance the classical SEIR model. Specifically, this model incorporates asymptomatic or mildly symptomatic infections and considers medical track quarantine, quarantine treatment, and other relevant measures in its construction. In order to capture the reality and complex dynamics of epidemic transmission more effectively, this model also incorporates distributed delays to define parameters such as contact rates and testing capabilities. Considering that certain parameters of the improved SEIR model require dynamic adjustments, regression algorithms and heuristic algorithms are utilized to calibrate these parameters, thereby enhancing the robustness of the model. The Italian epidemic (27, 28) experienced a second outbreak in August 2020. A survey by the Italian Business Association found that 21.1 million Italians went on holiday, accounting for 40% of the total population, which may have contributed to the epidemic’s resurgence (29). Thus, we analyze the Italian epidemic in two stages, with August 1st, 2020 as the cut-off point. The first stage pertains to the initial wave of the epidemic, while the second stage relates to the rebound after the resurgence. In this study, official announcement data from February to September 2020 was utilized. For each stage, a portion of the data was properly allocated as a training set to perform model parameter inversion. The data not included in the model training process was used to evaluate the predictive performance of the model and predicted the endpoint of the epidemic under these preventive measures. Additionally, sensitivity analysis experiments were conducted on the epidemic-related parameters in the model to analyze the impact of anti-epidemic measures on the spread of the disease.

## 2. Materials and methods

### 2.1. Model assumptions

It is essential to consider the divergence between the actual situation and the model. Therefore, this study incorporates the transmission characteristics of COVID-19 and makes several assumptions regarding the model, which are as follows:

1. Patients in the incubation period are able to infect susceptible individuals, while recovered patients are immune to reinfection due to the presence of antibodies. Furthermore, asymptomatic infections have a low mortality rate, and therefore, are not taken into consideration at present. Additionally, it is assumed that patients in quarantine areas are not infectious.
2. We have chosen the scenario of “closing the city” in China’s Hubei province as a template for our model. It should be noted that this model is structured as a closed node and does not account for population inflow/outflow or birth/death rates. While these factors may have an impact on the spread of COVID-19, they are not considered within the scope of this study.
3. Firstly, it is assumed that during the early stages of the outbreak, all residents complied with the government’s call to stay at home and wear masks while outside. Secondly, while most previous studies define the contact rate as a constant parameter, this does not accurately depict the dynamic implementation of anti-epidemic measures in different countries. It is recognized that not all infected individuals can be promptly detected. Considering that distributed delay plays a crucial role in capturing the time lag between infection, symptom onset, and subsequent transmission. Incorporating distributed delay in SEIR models helps to account for the variability observed in the incubation period, the time from infection to the development of symptoms, as well as other factors that influence disease progression and transmission (30). Therefore, Main novelty of this improved SEIR model is its ability to incorporate changes in contact rate c, detection capability T, and recovery rate through time varying deterministic and stochastic assumptions. The specific expressions utilized are outlined below:

(1) $$c=\left\{\begin{array}{c} \frac{\partial }{t+1}\\ \begin{array}{c} \;\\ b \end{array} \end{array}\right.\begin{array}{c} \;\\ \;\\ \; \end{array}\begin{array}{c} c>b\\ \;\\ c\leq b \end{array}$$

(2) $$T=\left\{\begin{array}{c} d_{1}\times t\\ d_{2}\times t\\ 1 \end{array}\right.\begin{array}{c} \;\\ \;\\ \; \end{array}\begin{array}{c} 0\leq t<n_{1}\\ n_{1}\leq t<n_{2}\\ n_{2}\leq t \end{array}$$

In the equation, ∂ represents the maximum contact rate at the initial stage, while b denotes the minimum contact rate. Due to successful implementation of prevention and control measures, the likelihood of infected individuals coming into contact with susceptibles is significantly reduced, thus we assume that b = 0. The coefficients d_1_ and d_2_, on the other hand, refer to the rate of change in detection capability.

During an epidemic, variations in medical treatment and human immunity can cause fluctuations in the daily recovery rate. In order to account for these fluctuations, we calculate the daily recovery rate using the following equation:


(3) $$Y=\frac{R\left(t\right)-R\left(t-1\right)}{I\left(t-1\right)}$$

R (t) represents the number of individuals who have recovered on a given day, while R (t-1) denotes the number of individuals who had recovered the previous day. Additionally, I (t-1) is the number of existing patients on the previous day.

To calculate the relationship between the daily recovery rate and time during an epidemic, we utilize Eq. (3) to determine the actual daily recovery rate. Next, we establish the following regression equation:


(4) $$\gamma =\varepsilon_{0}+\varepsilon_{1}\times t+\varepsilon_{2}\times t^{2}$$

The regression coefficients $\varepsilon_{0}$, $\varepsilon_{1}$, $\varepsilon_{2}$ are calculated by gradient descent method.

### 2.2. Improvement of SEIR model

In the traditional SEIR model, there are four categories of individuals: susceptible (S), exposed (E), infected (I), and recovered (R). However, this model has limitations in accurately simulating the actual epidemic spread. To address these limitations, we have improved the model by dividing the infected (I) category into two subcategories: asymptomatic infected (A) and symptomatic infected (I), as well as hospitalized (H) and deceased (D). Additionally, given the government’s measures to track the contacts of infected individuals, we further divided those in close contact with infected individuals into two subcategories: quarantined susceptible (S_q_) and quarantined exposed (E_q_).

**Figure 1:**
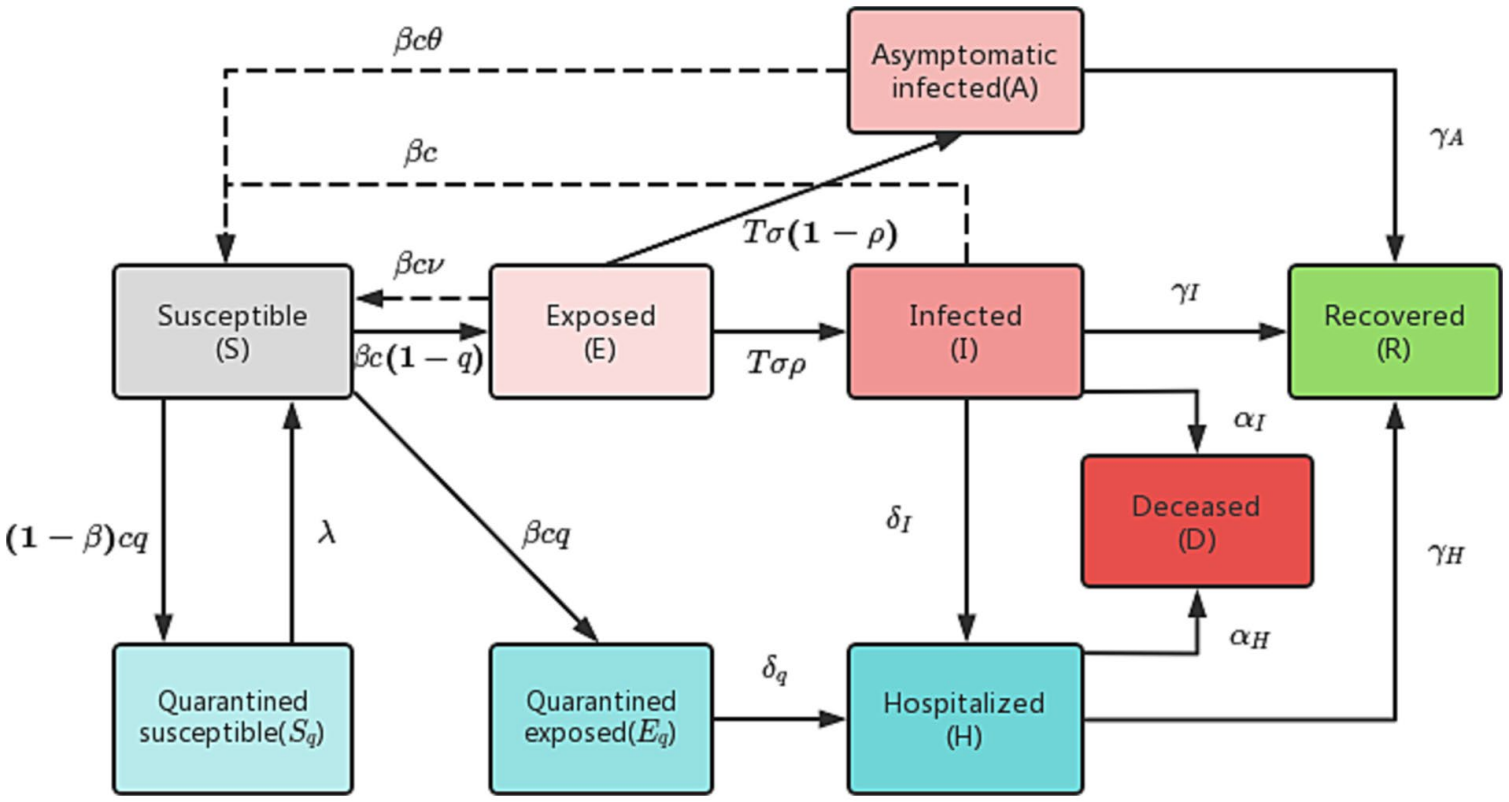
Population transformation diagram of the improved SEIR model.

In our improved model, we define q as the track quarantine ratio, β as the probability of infection, and c as the contact rate. Close contacts are quarantined at a rate of q, and if they become infected, they are transferred to the quarantined exposed (E_q_) category at a rate of βcq. Otherwise, they will be transferred to the quarantined susceptible (S_q_) category at a rate of $\left(1-\beta \right)cq$. Quarantined exposed individuals (E_q_) are then transferred to hospital quarantine treatment at a rate of $\delta_{q}$, while quarantined susceptible individuals (S_q_) are removed from quarantine and moved into the susceptible (S) category at a rate of λ. The population transformation relationships are illustrated in Figure 1.

An improved differential equation of SEIR transmission dynamics is as follows:


(5) $$\left\{ \begin{array}{l} \frac{dS}{dt}=-\left(\beta c+cq\left(1-\beta \right)\right)\frac{S}{N}\left(I+\theta A+\upsilon E\right)+\lambda S_{q}\\ \frac{dE}{dt}=\beta c\left(1-q\right)\frac{S}{N}\left(I+\theta A+\upsilon E\right)-T\sigma E\\ \frac{dI}{dt}=T\sigma \rho E-\left(\delta_{I}+\alpha_{I}+\gamma_{I}\right)I\\ \frac{dA}{dt}=T\sigma \left(1-\rho \right)E-\gamma_{A}A\\ \frac{dS_{q}}{dt}=\left(1-\beta \right)cq\frac{S}{N}\left(I+\theta A+\upsilon E\right)-\lambda S_{q}\\ \frac{dE_{q}}{dt}=\beta cq\frac{S}{N}\left(I+\theta A+\upsilon E\right)-\delta_{q\;}E_{q}\\ \frac{dH}{dt}=\delta_{I}I+\delta_{q}E_{q}-\left(\alpha_{H}+\gamma_{H}\right)H\\ \frac{dR}{dt}=\gamma_{I}I+\gamma_{A}A+\gamma_{H}H\\ \frac{dD}{dt}=\alpha_{H}H+\alpha_{I}I \end{array}\right.$$

Parameter settings are shown in Table 1.

**Table 1:** Improved SEIR transmission dynamics differential equation parameter table.

Parameter	Definition	Value	Source
β	infectious rate	0.099	Estimated
q	track quarantine ratio	0.5	Estimated
c	contact rate	Eq. (1)	Estimated
∂	initial maximum contact rate	28.3	Estimated
λ	quarantined release rate	1/14	References (13)
υ	the regulator of infectious probability in exposed	0.692	Estimated
σ	conversion rate from exposed to infected	1/7	References (12)
T	detection capability	Eq. (2)	Estimated
θ	the regulator of infectious probability in asymptomatic infected	0.88	Estimated
ρ	probability of symptomatic infected	0.14	References (15)
$\delta_{I}$	quarantined rate of symptomatic infected	0.13	References (12)
$\delta_{q}$	Conversion rate from quarantine exposed to quarantine healer	0.13	References (12)
$\alpha_{I}$	death rate of symptomatic infected	0.1	Estimated
$\alpha_{H}$	death rate of patients in quarantine treatment	0.0065	Estimated
$\gamma_{I}$	recovery rate of symptomatic infected	Eq. (10)	Estimated
$\gamma_{A}$	recovery rate of asymptomatic infected	Eq. (10)	Estimated
$\gamma_{H}$	recovery rate of patients treated in hospitalized	Eq. (10)	Estimated

### 2.3. Parameter estimation

Since the improved SEIR model relies on multiple parameter specifications provided in Table 1, with some parameters being dynamic and exhibiting significant variations across different states, the crucial task is to calibrate the relevant parameters within the model (31, 32). The primary objective is to ensure that the improved SEIR model is suitable for data from any given state. To achieve this goal, we utilize both regression algorithms and heuristic algorithms to estimate the model parameters based on actual data from February to May 2020.

#### 2.3.1. The regression algorithm fits the daily recovery rate

The regression algorithm is a widely used supervised learning algorithm in machine learning. In our study, we employ the gradient descent algorithm to solve for the regression coefficients and identify the nonlinear relationship between the number of days and the daily recovery rate. Specifically, we assume that the functional relationship between time and recovery rate can be represented by the following equation:


(6) $$\gamma =\varepsilon_{0}+\varepsilon_{1}\times t+\varepsilon_{2}\times t^{2}$$

Within our equation, t represents the number of days, and $E_{0}$, $E_{1}$, and $E_{2}$ denote the regression coefficients. These coefficients are used to estimate the nonlinear relationship between time and the daily recovery rate.

In our study, we utilize the mean square error criterion to establish an expression for the loss function. This criterion is defined as the average of the squared distances between the predicted values and the actual values. By employing this criterion, we can accurately measure the deviation between predicted and actual values in our model.


(7) $$J\left(\varepsilon \right)=\frac{1}{2m}\sum_{i=1}^{m}{\left(Y\left(t\right)-\gamma \left(t\right)\right)}^{2}$$

Where $J\left(\varepsilon \right)$ is the loss function, $Y\left(t\right)$ is the sample observation value, $\gamma \left(t\right)$ is the prediction value, and k is the number of data sets.

We derive the partial derivation of the loss function $J\left(\varepsilon \right)$:


(8) $$\nabla J\left(\varepsilon \right)=\frac{1}{m}\sum_{i=1}^{m}\left(Y\left(t\right)-\gamma \left(t\right)\right)\times P\left(j\right)$$

The $P\left(j\right)$ is the independent variable corresponding to the j coefficient.

Then we initialize ε randomly, and then iterate along the direction of negative gradient, so that the updated ε makes $J\left(\varepsilon \right)$ smaller. The equation is as follows:


(9) $$\varepsilon_{j}=\varepsilon_{j}-\alpha \times \sum_{i=1}^{m}\left(Y\left(t\right)-\gamma \left(t\right)\right)\times P\left(j\right)$$

**Figure 2:**
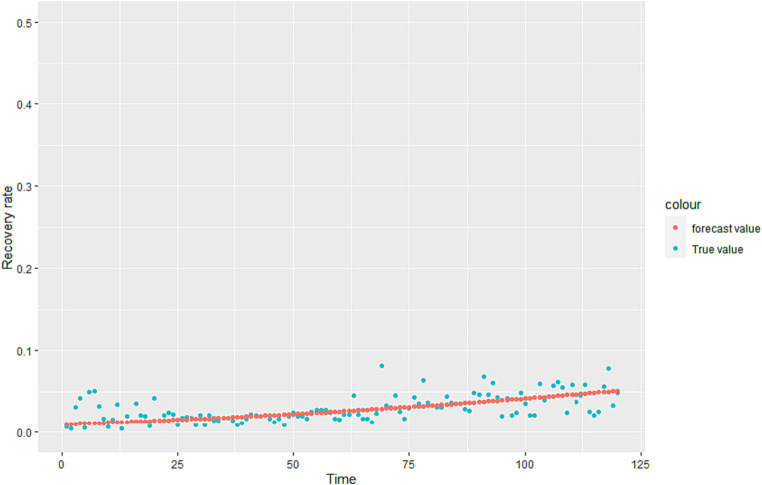
Fitting effect graph based on Italian daily recovery rate data.

Where α is the learning rate. When $\varepsilon_{j}$ drops to a certain point or a defined minimum value, it stops descending, and substitutes the $\varepsilon_{j}$ into the loss function to get the minimum value. The regression coefficient is estimated and the regression equation of γ is obtained. The fitting results are shown in Figure 2.

We calculated that γ is the overall recovery rate, but the recovery rate of the infected (I) and the infected (A) is different in reality. Therefore, we give reasonable assumptions based on the fitting effect of the model.

Assuming $\gamma_{A}=1.5\;\;\times \;\;\gamma_{I}$, $\gamma_{I}=\gamma_{H}$, according to the ratio of the number of infected (I) to infected (A), the results can be calculated:


(10) $$\left\{\begin{array}{c} \gamma_{I}\\ \gamma_{A}\\ \gamma_{H} \end{array}\begin{array}{c} =\\ =\\ = \end{array}\right.\begin{array}{c} 0.71\;\;\times \;\;\gamma \\ 1.05\;\;\times \;\;\gamma \\ 0.71\;\;\times \;\;\gamma \end{array}$$

#### 2.3.2. Heuristic algorithm to obtain model unknown parameters

Heuristic algorithms are applied to numerous real-world problems, allowing for the approximation of effective solutions in situations where guaranteeing the optimal solution is not feasible. Using heuristic-based models has contributed to a better understanding of the transmission trajectory of epidemics and increased the reliability of simulations (33). In this study, the Genetic Algorithm (GA) was utilized to calibrate the relevant parameters within the model. The objective of the algorithm is to optimize specific state-dependent parameters in order to enhance the agreement between the model and data. Each set of parameters for the SEIR model, along with the corresponding results, is referred to as an “agent.” Each agent possesses a unique input set of state-dependent parameters, which serves as its genome. The genome is comprised by a list of the state-dependent parameters: initial maximum contact rate (∂), infection rate (β), track quarantine ratio (q), death rate of symptomatic infection ($\alpha_{I}$), death rate of patients in quarantine treatment ($\alpha_{H}$), the ratio of the propagation capacity of exposed to infected (υ), and the regulator of infectious probability in asymptomatic infected (θ).

In order to optimize the parameters using a genetic algorithm, the agents’ performance is evaluated based on their “fitness,” which measures how well the agent matches the available data. In this study, fitness is determined by comparing the SEIR model’s results with the available data on cases, recoveries, and deaths. The fitness value is calculated using Eq. (11).


(11) $$\begin{array}{l} \text{Min}\;\left(U\left(\omega \right)\right)=\frac{1}{t}\sum_{i=1}^{t}{\left(I\left(t\right)=A\left(t\right)+H\left(t\right)+D\left(t\right)+R\left(t\right)-Z_{1}\right)}^{2}\\ \;+\;\;{\left(R\left(t\right)-Z_{2}\right)}^{2}+{\left(D\left(t\right)-Z_{3}\right)}^{2} \end{array}$$

$Z_{1}$ is the official daily number of cumulative infected cases, $Z_{2}$ is the number of cumulative recovered cases, $Z_{3}$ is the number of cumulative deaths cases.

Specifically, we set value ranges and sampling intervals for the unknown parameter set $\omega =\left\{\partial ,\;\;\mathrm{q,}\;\;\beta ,\;\alpha_{I},\;\;\alpha_{H},\;\;\upsilon ,\;\;\theta \right\}$ and utilized a genetic algorithm to randomly sample the parameters. The sampled values were then brought into the modified SEIR model, generating 2000 Monte Carlo simulations from the model. Using simulated data of the parameters as samples, we obtained the 95% confidence interval as the new sampling interval. Following further sampling using the genetic algorithm within the revised parameter intervals, the optimal value of the parameter set ω is revealed in Table 2.

**Table 2:** Confidence interval and values of parameters.

Parameter	95%CI	Value
initial maximum contact rate (∂)	(27.5–28.7)	28.3
infection rate (β)	(0.098–0.102)	0.099
track quarantine ratio (q)	(0.493–0.511)	0.5
death rate of symptomatic infection ($\alpha_{I}$)	(0.098–0.139)	0.1
death rate of patients in quarantine treatment ($\alpha_{H}$)	(0.0064–0.0074)	0.0065
the ratio of the propagation capacity of exposed to infected (υ)	(0.687–0.696)	0.692
the regulator of infectious probability in asymptomatic infected (*θ*)	(0.872–0.885)	0.88

## 3. Model verification and analysis

### 3.1. Model verification

**Figure 3:**
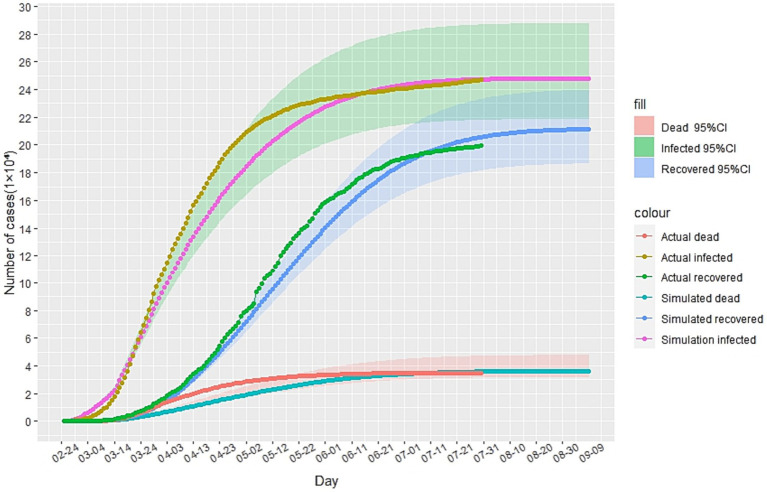
The simulation results of the epidemic situation in Italy without a second outbreak.

In this study, we utilized China’s Hubei anti-epidemic methods as a reference to analyze and predict the second outbreak of the Italian epidemic. Due to the gradual stabilization of the COVID-19 situation in Italy in July 2020, this study first simulated and predicted the hypothesis that there will be no second outbreak of the COVID-19 in Italy. The cases recorded from February to May 2020 were selected as the training set to calibrate the parameters of the improved SEIR model, while the cases from June to July were used as data not used for model fitting to evaluate the predictive performance of the SEIR model. The analysis results, as depicted in Figure 3, indicate that in the absence of a second outbreak, the epidemic was largely under control by late July 2020. Based on our model prediction, the peak number of cumulative infected cases would be approximately 250,000 (95%CI: 218,000-287,000), the peak number of cumulative recovered cases would be around 210,000(95%CI: 186,000-239,000), and the peak number of cumulative deaths would be approximately 36,000(95%CI: 31,700-41,600). Furthermore, since February 24, 2020, the actual daily cumulative number of reported cases in Italy has been mostly within the 95% confidence interval of the model simulation results, highlighting the accuracy of our simulation and prediction.

**Figure 4:**
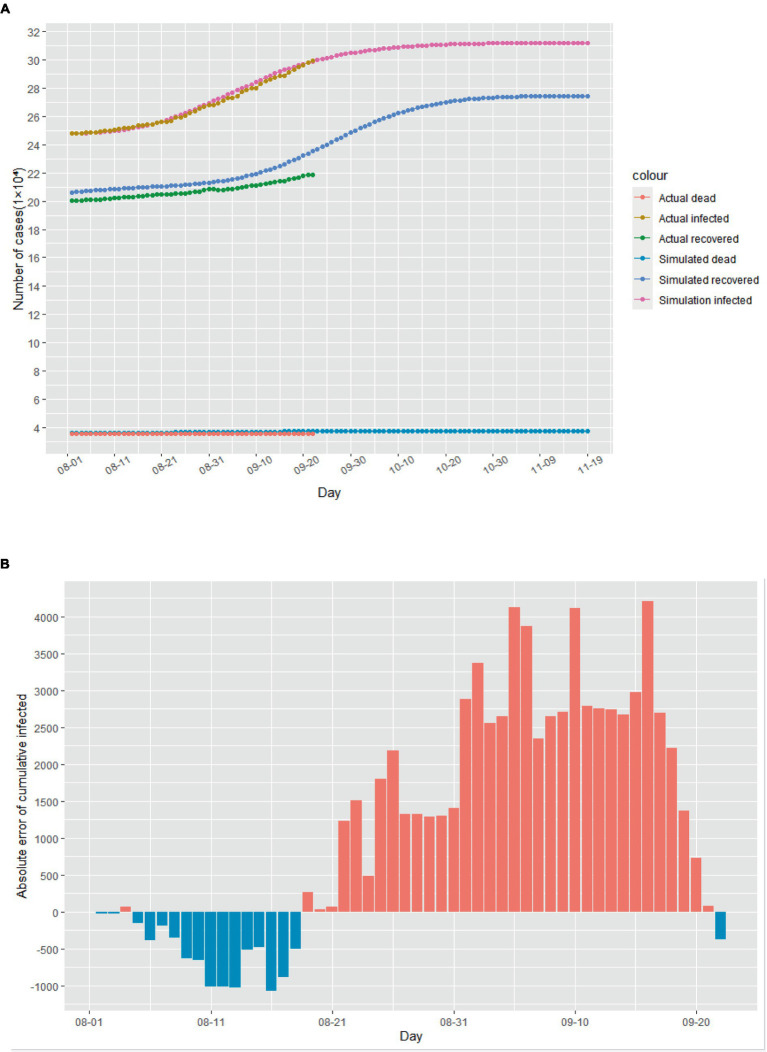

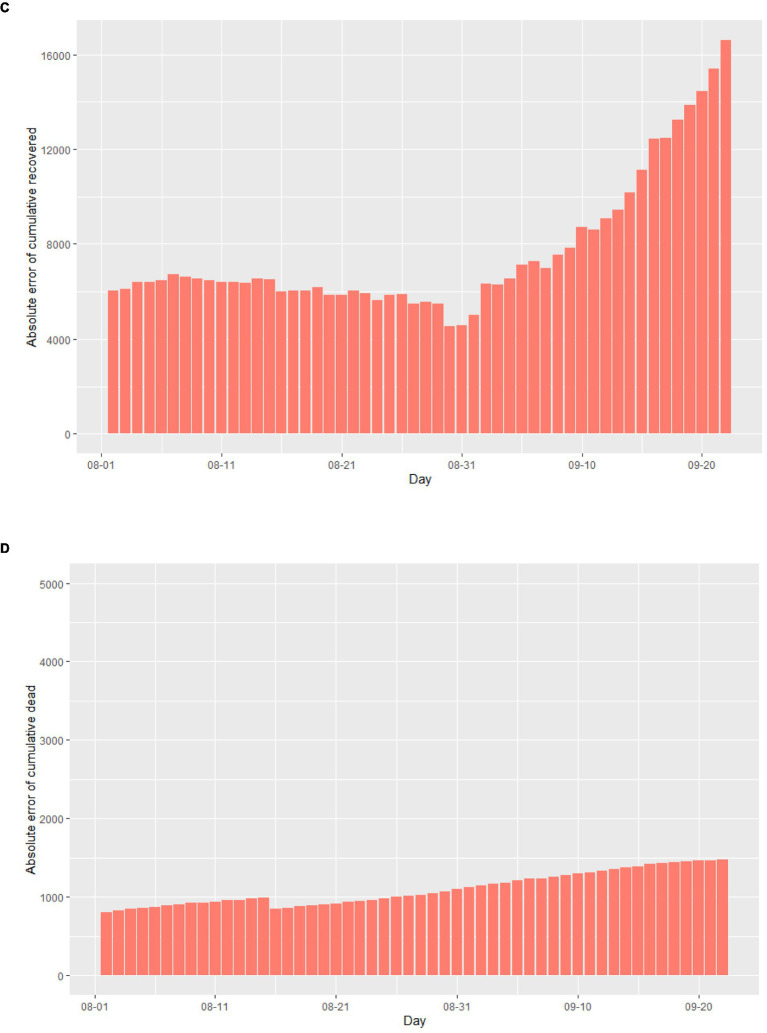
The simulation results of the epidemic situation in the case of a second outbreak in Italy. **(A)** Simulation and prediction of the second outbreak in Italy. **(B)** Absolute error of cumulative infected cases. **(C)** Absolute error of cumulative recovered cases. **(D)** Absolute error of cumulative dead cases.

Assuming that August 1st, 2020 marked the beginning of the second outbreak, we utilized the above model to conduct simulations from that day onwards. Due to intensified government anti-epidemic measures and changes in public perception compared to the initial phase of the epidemic, we refitted the model parameters using actual cases collected in August 2020. The cases collected in September were used as data not used for model fitting to evaluate the predictive performance of the SEIR model. The corrected parameters were then incorporated into the model. As illustrated in Figure 4A, according to this simulation model, the Italian epidemic situation would be largely under control by late October 2020, with the number of cumulative infected cases peaking on November 1st, 2020. Additionally, Figures 4B–D demonstrate the absolute error existing between the simulated data and actual data.

Based on the simulated and predicted results of the epidemic during the two mentioned time periods, the improved SEIR model eventually converges to a stable equilibrium state. This implies that as time progresses, the population sizes of each group reach a steady value, with no significant changes occurring. In this equilibrium state, the spread of the infectious disease is balanced with the rate of recovery or immunity, resulting in either the cessation of transmission or maintaining it at a low level within the population (34). This also indicates the existence of global solutions within this model (35).

### 3.2. The impact of anti-epidemic measures on the spread of the epidemic

**Figure 5:**
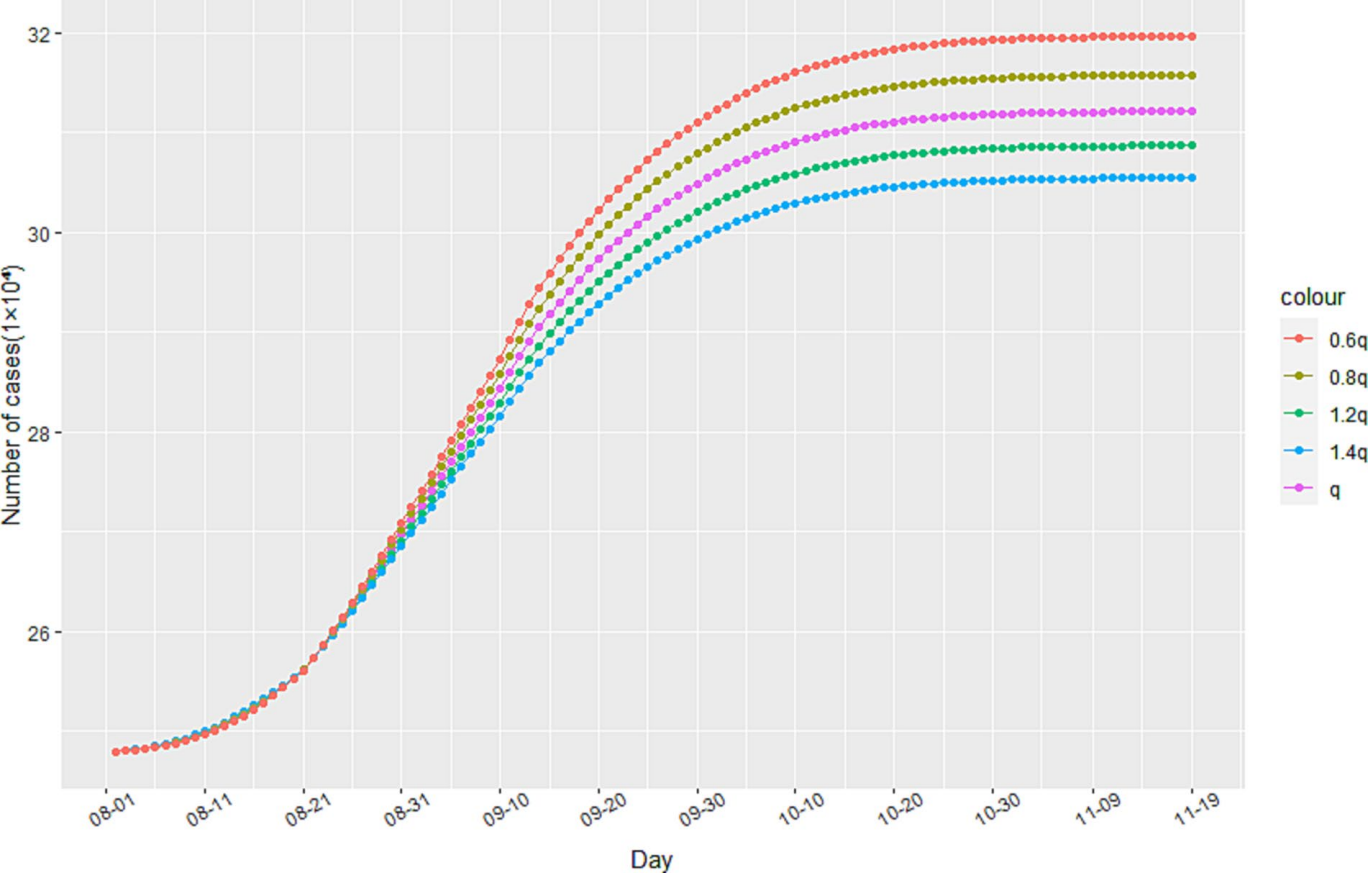
The impact of track quarantine on the spread of the epidemic.

To analyze the impact of anti-epidemic measures on the trend of the epidemic, we employ sensitivity analysis experiments within our model. Specifically, we observe the development of the epidemic in Italy since August 1st, 2020 through changes in the track quarantine ratio q. As illustrated in Figure 5, reductions in the proportion of track quarantine to 0.8q and 0.6q resulted in increases in the number of cumulative infections. Conversely, when the proportion of track quarantine was increased to 1.2q and 1.4q, the number of cumulative infections decreased accordingly. These findings demonstrate that strict medical track quarantine is an effective means of preventing the spread of the epidemic.

As government interventions on the epidemic become more stringent, residents are encouraged to stay at home, reduce their number of trips, and practice personal protection measures. This results in a decrease in the dynamic variable for the contact rate of infected individuals, as shown in Eq. (1). The minimum contact rate b can be expressed as the final steady state of the infected persons’ contact rate under anti-epidemic measures. Given that this model is based on China’s strict anti-epidemic measures, we assume that the minimum contact rate is *b* = 0. However, it is important to consider the impact of changes in b on the epidemic under different anti-epidemic measures, as not all countries can implement measures as strict as China’s.

**Figure 6:**
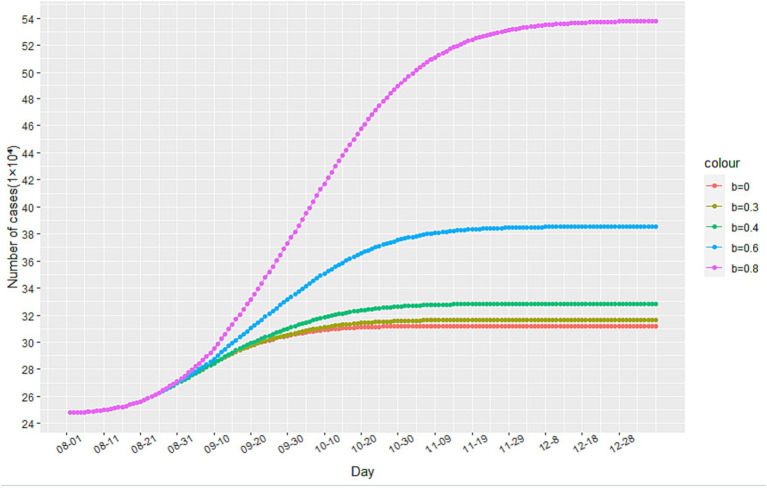
The impact of changes in the minimum contact rate b on the number of cumulative infected cases.

**Figure 7:**
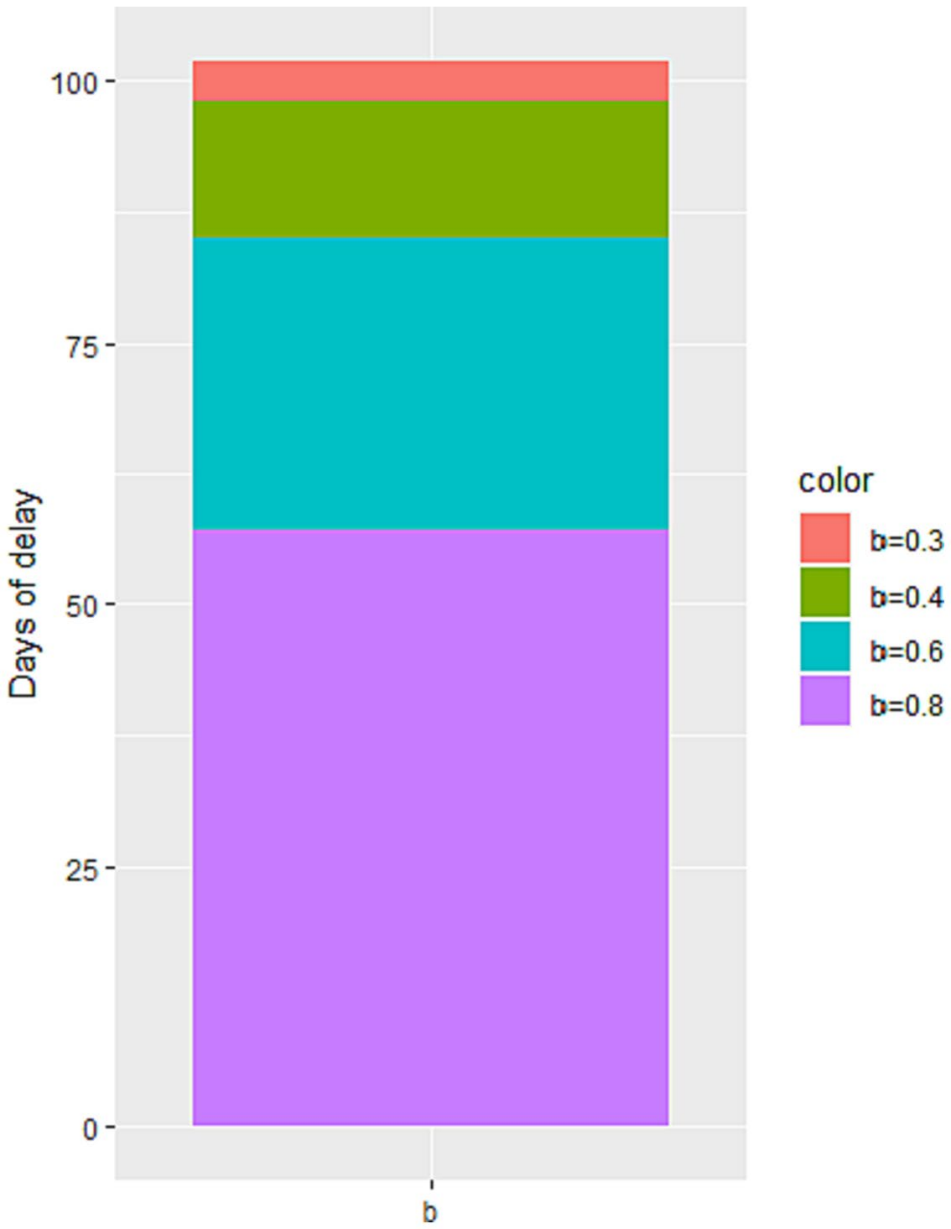
The impact of the change in the minimum contact rate b on the peak time of the number of cumulative infected cases.

Through simulation, we find that increasing the value of b leads to an increase in the number of cumulative infected cases during the second stage of the epidemic, as illustrated in Figure 6. When b reaches 0.6 and 0.8, there is a significant increase in the number of cumulative infected cases. However, when b equals 0.3, the increase is less noticeable. These findings suggest that reducing the flow of people can achieve a similar effect to a completely enclosed quarantine. Additionally, Figure 7 demonstrates that the time taken to reach the peak number of cumulative infected cases also increases with the rise of b. Therefore, by strengthening anti-epidemic measures and reducing population movement, it is possible to effectively lower the peak number of infections and bring the epidemic under control earlier.

## 4. Conclusion

This study proposes an improved SEIR epidemiological model to retrospectively analyze and predict the transmission trajectory of COVID-19 in Italy from February to September 2020. In this model, besides considering specific states for different population groups, dynamic parameter settings are incorporated by adding time varying deterministic and stochastic assumptions. This enables the model to better capture the realistic and complex dynamics of the epidemic spread. It is worth noting that some parameters of the improved SEIR model exhibit significant variations across different states. Therefore, regression algorithms and heuristic algorithms are used to calibrate these parameters, making the model applicable to data from any given state. By simulating and predicting the transmission trajectories during two periods, before and after the secondary outbreak in Italy, the numerical results demonstrate that the model possesses good long-term predictive performance and robustness. The model was then utilized to analyze the impact of anti-epidemic measures on the epidemic’s development and predict future trends. The results indicate that:

1. Strict medical track quarantine is effective in restraining the epidemic’s development.
2. Reducing population movement, practicing personal protection, and avoiding contact with infected individuals can effectively reduce the peak number of infections, leading to earlier control of the epidemic.
3. Lowering the proportion of population movement can achieve similar outcomes to complete lockdowns.

To curb the epidemic’s growth, Italy must enhance anti-epidemic measures, reduce population movement, and promote public awareness of epidemic prevention measures to maintain the minimum contact rate of infected individuals within a small range.

As this model was based on a closed space, it did not account for the inflow and outflow of populations between Italy and other countries. Thus, we plan to conduct further research by launching SEIR model simulation studies that take into consideration transportation, temporal and spatial distribution, population movement, and multiple patches. Through these analyses, we aim to develop an epidemic spread prediction and precise prevention control system based on temporal and spatial big data. This will provide more comprehensive insights into the dynamic nature of COVID-19 transmission, allowing authorities to formulate more effective measures to prevent and curb its spread.

## Data availability statement

The original contributions presented in the study are included in the article/Supplementary material, further inquiries can be directed to the corresponding author.

## Author contributions

ML and X-yZ: data collection, investigation, data analysis, and writing—original draft and revision. W-nJ: data collection and writing. C-zT: conceptualization. All authors contributed to the article and approved the submitted version.

## Conflict of interest

The authors declare that the research was conducted in the absence of any commercial or financial relationships that could be construed as a potential conflict of interest.

## Publisher’s note

All claims expressed in this article are solely those of the authors and do not necessarily represent those of their affiliated organizations, or those of the publisher, the editors and the reviewers. Any product that may be evaluated in this article, or claim that may be made by its manufacturer, is not guaranteed or endorsed by the publisher.
